# Microsurgical reconstruction in midface defects based on defect morphology and size – Reconstruction techniques, complications, and outcomes

**DOI:** 10.1007/s10006-026-01566-0

**Published:** 2026-05-09

**Authors:** Felix Neumann, Phillip Gomme, Jakob Fenske, Philipp Lampert, Oliver Wagendorf, Friedrich Mrosk, Elena Hofmann, Susanne Nahles, Max Heiland, Kilian Kreutzer, Anna-Christina Sofroniou, Christian Doll, Steffen Koerdt

**Affiliations:** 1 Department of Oral and Maxillofacial Surgery, Charité – Universitätsmedizin Berlin, Corporate Member of Freie Universität Berlin and Humboldt-Universität Zu Berlin, Augustenburger Platz 1, 13353 Berlin, Germany; 2 Berlin Institute of Health at Charité – Universitätsmedizin Berlin, Charitéplatz 1, 10117 Berlin, Germany

**Keywords:** Head and neck cancer, Brown classification, Midface reconstruction, Microvascular reconstruction, Dental rehabilitation

## Abstract

**Purpose:**

This study´s aim was to analyse clinical outcomes of microvascular reconstructions performed for malignancy-caused midface defects of varying sizes at our department.

**Methods:**

This retrospective study included 98 patients (38 women; 60 men; age range 16–94 years) who underwent microvascular reconstruction for a malignancy-related midface defect between April 2017 and August 2022.

**Results:**

Bony (fibula, scapula) and soft tissue (radial forearm, anterolateral thigh) flap techniques were used depending on defect size. Most defects requiring coverage were classified as class II (*n = *70, 71.4%; 18 (25.7%) bony vs. 52 (74.3%) soft tissue) or class IV (*n = *16, 16.3%; 4 (25.0%) bony vs. 12 (75.0%) soft tissue) according to Brown and Shaw. The proportion of other horizontal defect classes was significantly lower (*n = *12, 12.2%; all soft tissue). Unilateral defects were present in 81 cases (82.7%; 15 (18.5%) bony vs. 66 (81.5%) soft tissue), while bilateral defects were observed in 17 cases (17.3%; 7 (42.2%) bony vs. 10 (57.8%) soft tissue). Postoperative complications were observed in 57 cases (58.2%), predominantly comprising minor issues such as bleeding or wound healing disorders. Major complications, including the need for transplant revisions, occurred in 9 cases (9.2%), with early transplant loss in three instances. No significant correlation was found between transplant loss and defect classification (*p = *0.73) or transplant type (*p = *0.07). However, bony flaps demonstrated a higher tendency towards flap loss compared to soft tissue flaps (18.2% vs 4.0%). Dental rehabilitation using dental implants was performed in only 18.2% of all patients who received bony reconstruction.

**Conclusion:**

The complication rate associated with microvascular reconstructions was relatively high. Most complications were minor and could be managed therapeutically. Major complications, such as flap failure, remained relatively uncommon.

Neither defect size nor transplant type used demonstrated a significant impact, though bony transplants showed a higher tendency towards flap loss.

Despite the observed complication rate, free flaps continue to represent a reliable treatment option for patients with midfacial defects. In cases where bony reconstruction is not strictly necessary, dental rehabilitation using patient-specific preprosthetic implants may offer a viable alternative to bone grafts.

## Introduction

The midface is one of the most complex regions in head and neck surgery, playing a crucial role in both aesthetics and function. Its intricate anatomy serves multiple essential functions while also having a significant impact on facial appearance. Consequently, midface reconstruction requires advanced surgical techniques that address both functional and aesthetic demands [1–4].

A primary indication for midface reconstruction is malignant disease. Surgical tumour resection often results in defects that necessitate reconstruction to restore function and aesthetics [5, 6]. The most common malignancy in the head and neck region remains squamous cell carcinoma, though other tumour entities, such as adenocarcinomas and melanomas, are also frequently encountered [7].

The classification of midface defects remains a topic of debate. One widely accepted system was introduced by Brown et al. in 2010. This classification includes both orbitomaxillary and nasomaxillary defects, categorising them based on vertical and horizontal aspects [8]:

Vertical classification:

I: Maxillectomy without oronasal fistula
II: Maxillectomy without involving the orbit
III: Maxillectomy involving the orbital adnexae with orbital retention
IV: With orbital enucleation or exenteratio
V: Orbitomaxillary defect
VI: Nasomaxillary defect

Horizontal classification:

a: palatal defect only
b: ≤ 50% unilateral
c: ≤ 50% bilateral or transverse anterior
d: ≥ hemimaxillectomy

Traditionally, midface reconstruction was performed using an obturator prosthesis, providing a reliable, single-stage solution that allows for dental rehabilitation, oronasal separation, and aesthetic restoration [9, 10].

Today, microvascular free flaps are widely regarded as the gold standard in reconstructive head and neck surgery. In addition, computer-aided design and manufacturing (CAD/CAM) technology enables precise preoperative planning, particularly for bony reconstructions, leading to improved accuracy and predictability. Free flap surgery offers a more anatomical and natural reconstruction, enhancing both function and aesthetics [3, 6, 11, 12].

Furthermore, patient-related factors such as alcohol consumption, smoking, diabetes mellitus (DM), and irregular body mass index (BMI) are known to influence wound healing in various ways [13–16]. Therefore, this study also evaluates their impact on postoperative complications in this specific patient cohort.

The aim of this study was to assess the outcomes of microvascular free flap reconstruction for midface defects caused by malignant tumours. Additionally, the study examines the applicability of different free flaps to various defect classes based on Brown’s classification. Furthermore, the difference between bony and soft tissue reconstructions are evaluated with respect to functional rehabilitation. Finally, potential risk factors and postoperative complications are analysed and an algorithm for reconstructive surgery in different midface defects, based on our surgical experience, is provided.

## Material and methods

This retrospective study analyses a cohort of patients with midface defects caused by malignant tumours, who underwent surgical reconstruction via microvascular free flap surgery at the Department of Oral and Maxillofacial Surgery at Charité Universitätsmedizin Berlin between April 2017 and August 2022.

Inclusion criteria:

I: Surgical resection of malignant tumours of the midface
II: Midface defect classifiable according to Brown’s classification
III: Reconstructive surgery via microvascular free flap

**Fig. 1:**
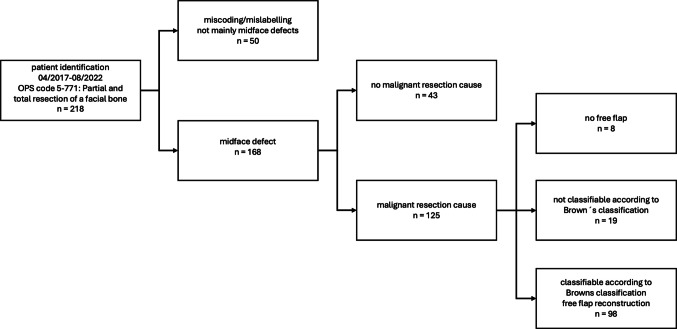
Patient selection workflow

The patient selection workflow is illustrated in Fig. 1.

Reconstructive surgery was performed either simultaneously with tumour resection or as a separate procedure. Patients were excluded if they had midface defects caused by non-malignant conditions, defects not classifiable by Brown’s classification, or if they had not undergone free flap reconstruction. All patients received postoperative anticoagulation therapy, either with fraxiparine or unfractionated heparin. Only patients with complete datasets were included.

### Statistical analysis

Data collection was conducted using Microsoft Excel (Microsoft Corporation, Redmond, WA, USA) and subsequently transferred to SPSS Statistics version 30 (IBM Corporation, Armonk, NY, USA) for further statistical analysis. Descriptive statistics were used to determine means and standard deviations (SD) as well as medians and first (Q1) and third (Q3) quartiles for metric variables. Absolute and relative frequencies were calculated for categorical data. Binomial logistic regression analysis was performed to assess correlations between clinical parameters and complications or flap loss. Statistical significance was defined as *p* < 0.05.

## Results

### Patient´s characteristics

This retrospective study included 98 patients (60 men [61.2%] and 38 women [38.8%]), all of whom underwent midface reconstructive surgery due to defects caused by malignant tumours of the midface. The mean age ± SD was 67.8 ± 17.0 years (range: 16–94 years, median: 73 years [Q1 = 61.8, Q3 = 80.0]). In 80 cases, the tumors were primary, while 6 cases involved secondary carcinomas. In 12 cases, reconstruction was preceded by treatment for recurrent disease. Data on tumour type and TNM classification are provided in Table 1.

**Table 1:** Tumour types of the study cohort

	N	%
Tumour type		
Oral squamous cell carcinoma (OSCC) Adenoid cystic carcinoma (ACC) Sarcoma Basal cell carcinoma (BCC) Melanoma Other	72 10 6 3 2 5	73.5 10.2 6.1 3.1 2.0 5.1

### Defect and reconstructive surgery

Flap surgery was performed in all cases, with the choice of flap type based on the defect characteristics, the patient’s physiological condition, and the surgeon’s preference. Tumor resection and flap reconstruction were performed in a single-stage procedure in 70 cases, whereas in 28 cases reconstruction was carried out as a separate, staged operation. The flap types used included fibula free flap (FFF; *n = *13, 13.3%; skin paddle in 9 cases and muscle without skin in 4 cases), scapula free flap (SFF; *n = *9, 9.2%; skin paddle in 7 cases an muscle without skin in 2 cases), radial forearm flap (RFF; *n = *50, 51.0%), anterolateral thigh flap (ALT; *n = *22, 22.4%; single skin paddle in 12 cases and muscle flap in 10 cases), latissimus dorsi myocutaneous flap (LDMF; *n = *2, 2.0%; both with skin paddle), parascapular free flap (PSFF; *n = *1, 1.0%; with skin paddle), and one latissimus dorsi–serratus anterior compound flap (LDSAF; *n = *1, 1.0%; with skin paddle). This resulted in 22 bony flaps (22.4%) and 76 soft tissue flaps (77.6%). Additionally, the region of the anastomosis was evaluated. In the vast majority of cases, the anastomosis was performed in the cervical region (95/98; 74 soft-tissue flaps and 21 bony flaps). In three cases, the anastomosis was carried out using preauricular vessels (two soft-tissue flaps and one bony flap). Vessel grafts were used in two cases. In one patient, a vein graft from the contralateral external jugular vein was used for the venous anastomosis. In the second case, a graft from the superior thyroid artery was used for the arterial anastomosis. In both cases, the grafts were required due to insufficient vessel length when performing the anastomosis in the cervical region. Complications were recorded in both groups (55/95 in the cervical group vs. 2/3 in the preauricular group). All flap losses occurred in the group with cervical anastomosis. There was no significant difference in the occurrence of complications between the two anastomotic locations (*p = *0.76). In 81 cases (82.7%), the defects were unilateral, while 17 cases (17.3%) involved bilateral defects. The Brown classification and corresponding flap types are summarised in Table 2.

**Table 2:** Overview of flap types, categorized by horizontal, vertical aspects and Brown classes

Brown classification			Flap type (n (%))						
			Bone		Soft tissue				
Horizontal aspect	n	%	FFF	SFF	RFF	ALT	LDMF	PSFF	LDSAF
I II III IV V	2 70 5 16 5	2.0 71.4 5.1 16.3 5.1	0 (0) 12 (17.2) 0 (0) 1 (6.3) 0 (0)	0 (0) 6 (8.6) 0 (0) 3 (18.8) 0 (0)	2 (100) 39 (55.7) 1 (20.0) 4 (25.0) 4 (80.0)	0 (0) 13 (18.6) 2 (40.0) 7 (43.8) 0 (0)	0 (0) 0 (0) 0 (0) 1 (6.3) 1 (20.0)	0 (0) 0 (0) 1 (20.0) 0 (0) 0 (0)	0 (0) 0 (0) 1 (20.0) 0 (0) 0 (0)
Vertical aspect									
A B C D	1 80 12 5	1.0 81.6 12.2 5.1	0 (0) 9 (11.3) 3 (25.0) 1 (20.0)	0 (0) 6 (7.5) 0 (0) 3 (60.0)	1 (100) 46 (57.5) 3 (25.0) 0 (0)	0 (0) 16 (20.0) 5 (41.7) 1 (20.0)	0 (0) 2 (2.5) 0 (0) 0 (0)	0 (0) 1 (1.3) 0 (0) 0 (0)	0 (0) 0 (0) 1 (8.3) 0 (0)
Classes									
I B II A II B II C II D III B III C IV B IV C IV D V B	2 1 58 9 2 3 2 12 1 3 5	2.0 1.0 59.2 9.2 2.0 3.1 2.0 12.2 1.0 3.1 5.1	0 (0) 0 (0) 8 (13.8) 3 (33.3) 1 (50.0) 0 (0) 0 (0) 1 (8.3) 0 (0) 0 (0) 0 (0)	0 (0) 0 (0) 5 (8.6) 0 (0) 1 (50.0) 0 (0) 0 (0) 1 (8.3) 0 (0) 2 (66.6) 0 (0)	2 (100) 1 (100) 35 (60.3) 3 (33.3) 0 (0) 1 (33.3) 0 (0) 4 (33.3) 0 (0) 0 (0) 4 (80.0)	0 (0) 0 (0) 10 (17.2) 3 (33.3) 0 (0) 1 (33.3) 1 (50.0) 5 (41.7) 1 (100) 1 (33.3) 0 (0)	0 (0) 0 (0) 0 (0) 0 (0) 0 (0) 0 (0) 0 (0) 1 (8.3) 0 (0) 0 (0) 1 (20.0)	0 (0) 0 (0) 0 (0) 0 (0) 0 (0) 1 (33.3) 0 (0) 0 (0) 0 (0) 0 (0) 0 (0)	0 (0) 0 (0) 0 (0) 0 (0) 0 (0) 0 (0) 1 (50.0) 0 (0) 0 (0) 0 (0) 0 (0)

### Complications and flap loss

Complications were observed across all flap types and defect classes, with overall 89 complication events in 57 cases (58.2%). The recorded complications included bleeding (*n = *14, 14.3% of all cases), partial necrosis (*n = *16, 16.3%), wound healing disorders (WHD, *n = *34, 34.7%), infections (*n = *8, 8.2%), bone exposure (BEx, *n = *1, 1.0%), flap revision (FRev, *n = *9, 9.2%), and flap loss (*n = *7, 7.1%). Bony flaps were affected by complications in 14 cases (63.6%), while soft tissue flaps were affected in 43 cases (56.6%). However, the difference between the two flap types was not statistically significant in the logistic regression analysis (*p = *0.56).

The impact of flap type on transplant loss was also assessed. Flap losses were recorded in fibula (2/12), scapula (2/9), radial (2/50), and ALT flaps (1/22). There was a tendency towards a higher incidence of flap loss in the bony flap group (*n = *4, 18.2%) compared to the soft tissue flaps (*n = *3, 4.0%), though this difference did not reach statistical significance (*p = *0.07). Additionally, it should be noted that only three of these cases were early flap losses, occurring within the first seven postoperative days. The remaining four flap losses took place later during the follow-up period. Flap revisions were performed in four soft tissue flaps (4/76; 5.3%) and five bony flaps (5/22; 22.7%). In two cases involving bony flaps and one case involving a soft tissue flap, the revision was unsuccessful, ultimately resulting in flap loss. This yields a successful flap revision rate of six out of nine cases (66.6%) in this cohort.

Additional clinical parameters and risk factors were analysed for their influence on complications. Neither sex nor age had a significant impact on the overall complication rate (sex: *p = *0.23; age: *p = *0.46) or the rate of flap loss (sex: *p = *0.48; age: *p = *0.22). Similarly, no statistically significant correlation was found between complications (including flap loss) and the risk factors of nicotine smoking, alcohol consumption, diabetes mellitus (DM), or irregular body mass index (BMI). A detailed evaluation of risk factors associated with complications is presented in Table 3.

**Table 3:** Complications categorised by risk factors

Risk factor			General complication (n (% of specific complication type))						
	n	%	Bleeding	Necrosis	WHD	Infection	BEx	FRev	
Alcohol	9	9.2	0 (0)	3 (18.8)	4 (11.8)	1 (12.5)	1 (100)	0 (0)	*p = *0.70
Nicotine	18	18.4	2 (14.3)	5 (31.3)	4 (11.8)	1 (12.5)	1 (100)	2 (22.2)	*p = *0.84
DM	14	14.3	2 (14.3)	4 (25.0)	4 (11.8)	1 (12.5)	1 (100)	1 (11.1)	*p = *0.71
BMI	50	51.0	8 (57.1)	9 (56.3)	13 (38.2)	4 (50.0)	1 (100)	7 (77.8)	*p = *0.89
			Flap loss (n (%of specific complication type))						
Alcohol	1	1.0	1 (14.3)						*p = *0.86
Nicotine	2	2.0	2 (28.6)						*p = *0.74
DM	1	1.0	1 (14.3)						*p = *0.90
BMI	4	4.1	4 (57.1)						*p = *0.79

Furthermore, the different Brown classes were analysed in relation to the prevalence of complications. Among the horizontal defect classes, class I exhibited complications in all cases (*n = *2, 100%), with flap loss occurring in one of the two cases (50.0%). In class II, complications were observed in 37 cases (52.9%), with four instances of flap loss (5.7%). In class III, complications occurred in four cases (80.0%), although no flap losses were recorded. In class IV, 12 cases (75.0%) experienced complications, including two cases of flap loss (12.5%). Class V presented complications in two cases (40.0%), though neither resulted in flap loss.

When analysed by vertical classification, complications were reported in one case in class A (100%), but no flap losses occurred (0%). In class B, complications were noted in 42 cases (52.5%), with five instances of flap loss (6.1%). In class C, 11 cases (91.7%) had complications, including two cases of flap loss (16.7%). In class D, complications were observed in three cases (60.0%), but no flap losses were reported (0%).

There was no statistically significant association between Brown classification and the prevalence of complications (*p = *0.35) or flap losses (*p = *0.73). A detailed summary of complications, categorised by horizontal and vertical defect classes, is presented in Table 4.

**Table 4:** Complications, categorised by horizontal, vertical and combined Brown classes

Brown classification			Complication (n (% of specific complication type))						
Horizontal	n	%	Bleeding	Necrosis	WHD	Infection	BEx	FRev	Flap loss
I II III IV V	2 70 5 16 5	2.0 71.4 5.1 16.3 5.1	1 (7.1) 8 (57.1) 1 (7.1) 3 (21.4) 1 (7.1)	0 (0) 8 (50.0) 3 (18.8) 4 (25.0) 1 (6.3)	1 (2.9) 22 (64.7) 2 (5.9) 9 (26.5) 0 (0)	0 (0) 3 (37.5) 0 (0) 5 (62.5) 0 (0)	0 (0) 0 (0) 0 (0) 1 (100) 0 (0)	0 (0) 8 (88.9) 0 (0) 1 (11.1) 0 (0)	1 (14.3) 4 (57.1) 0 (0) 2 (28.6) 0 (0)
Vertical									
A B C D	1 80 12 5	1.0 81.6 12.2 5.1	0 (0) 11 (78.6) 3 (21.4) 0 (0)	0 (0) 11 (68.8) 5 (31.3) 0 (0)	1 (2.9) 23 (67.6) 7 (20.6) 3 (8.8)	0 (0) 6 (75.0) 1 (12.5) 1 (12.5)	0 (0) 1 (100) 0 (0) 0 (0)	0 (0) 6 (66.7) 2 (22.2) 1 (11.1)	0 (0) 5 (71.4) 2 (28.6) 0 (0)
Combined									
I B II A II B II C II D III B III C IV B IV C IV D V B	2 1 58 9 2 3 2 12 1 3 5	2.0 1.0 59.2 9.2 2.0 3.1 2.0 12.2 1.0 3.1 5.1	1 (7.1) 0 (0) 5 (35.7) 3 (21.4) 0 (0) 1 (7.1) 0 (0) 3 (21.4) 0 (0) 0 (0) 1 (7.1)	0 (0) 0 (0) 6 (37.5) 2 (12.5) 0 (0) 1 (6.3) 2 (12.5) 3 (18.8) 1 (6.3) 0 (0) 1 (6.3)	1 (2.9) 1 (2.9) 15 (44.1) 5 (14.7) 1 (2.9) 1 (2.9) 1 (2.9) 6 (17.6) 1 (2.9) 2 (5.9) 0 (0)	0 (0) 0 (0) 2 (25.0) 1 (12.5) 0 (0) 0 (0) 0 (0) 4 (50.0) 0 (0) 1 (12.5) 0 (0)	0 (0) 0 (0) 0 (0) 0 (0) 0 (0) 0 (0) 0 (0) 1 (100) 0 (0) 0 (0) 0 (0)	0 (0) 0 (0) 6 (66.7) 2 (22.2) 0 (0) 0 (0) 0 (0) 0 (0) 0 (0) 1 (11.1) 0 (0)	1 (14.3) 0 (0) 3 (42.9) 1 (14.3) 0 (0) 0 (0) 0 (0) 1 (14.3) 1 (14.3) 0 (0) 0 (0)

### Dental rehabilitation

Dental rehabilitation using implants was achieved in four (18.2%) cases following bony flap surgery. One additional patient received preprosthetic maxillary implants. In the remaining cases, implant-based rehabilitation was not carried out due to individual patient factors, such as tumour recurrence, poor general health, inadequate oral hygiene, the need for adjuvant therapies, or a lack of patient willingness to proceed with implant treatment.

## Discussion

Midface reconstructive surgery should aim to achieve both aesthetic and functional restoration. Consequently, the chosen reconstruction method must fulfil key objectives, including the separation of intracranial and extracranial structures, orbital support, the restoration of orbital and dental function, and the reconstruction of midfacial aesthetics [2]. In modern oral and maxillofacial surgery, these objectives are often accomplished through microsurgical flap procedures, which are considered the gold standard for reconstructing extensive facial defects [17].

Although the Brown classification is widely accepted as a major system for categorising midfacial defects, its value for guiding reconstructive decision-making remains debated. Artopoulou et al. demonstrated that, although defect size influenced patients’ quality of life, it did not predict functional outcomes [18]. Similarly, a systematic review by Chandra and Chang highlighted the considerable variability in reconstructive approaches within individual Brown classes. In particular, within Class II—also the most common category in the present study—a wide range of reconstructive options is reported. Notably, the literature describes comparable functional outcomes between obturator prostheses and free flap reconstruction in terms of speech, swallowing, and mastication. This supports our findings, which demonstrate the use of both bony and soft tissue flaps within this group, thereby calling into question the validity of the Brown classification as a predictor for selecting the most appropriate reconstructive method [19].

Consequently, the Brown classification should be regarded primarily as a descriptive rather than a predictive system for midfacial defects. In response to these limitations, a more comprehensive framework has recently been proposed by Mommaerts et al., incorporating both vertical and horizontal dimensions alongside functional parameters, such as alveolar involvement and a more precise delineation of horizontal defect extent. This approach better reflects the complexity of midfacial defects and may prove valuable in future clinical practice, although it has not yet been widely adopted [20].

Vertical aspects:

- A1: only alveolar process involved
- A2: cranial extension into maxilla with possible nasal involvement but no orbital involvement
- A3: involvement of the orbital floor
- A4: A3 + enucleation
- A5: A4 + dura mater exposure
- B1: no alveolar involvement, middle third/nasal area affected
- B2: malignancy reached the orbital floor
- B3: B2 + enucleation
- B4: B3 + dura mater exposure

The modifier *m* can be appended for mandibular involvement.

Horizontal aspects:

- Solitary internal defect
- Partial unilateral involvement
- H3 unilateral defect extending to the midline
- H4 defect crosses the midline (bilateral)
- H5 entire horizontal dimension of the upper jaw

In our study, RFFs were the most frequently used in midface reconstruction. This aligns with current literature, which identifies the RFF as the preferred free flap for midface reconstruction, particularly for smaller defects that do not require urgent bony reconstruction. Some authors also advocate the use of osteocutaneous RFFs to incorporate bony reconstruction [2, 3, 11]. A key advantage of using RFFs in midface reconstruction is their suitability for cases requiring a long vascular pedicle, given the significant distance between the midface and cervical vessels that must be bridged [2, 21]. However, at our centre, other osseous flaps, such as the fibula free flap (FFF) and scapula free flap (SFF), are favoured for bony maxillary reconstruction.

**Fig. 2:**
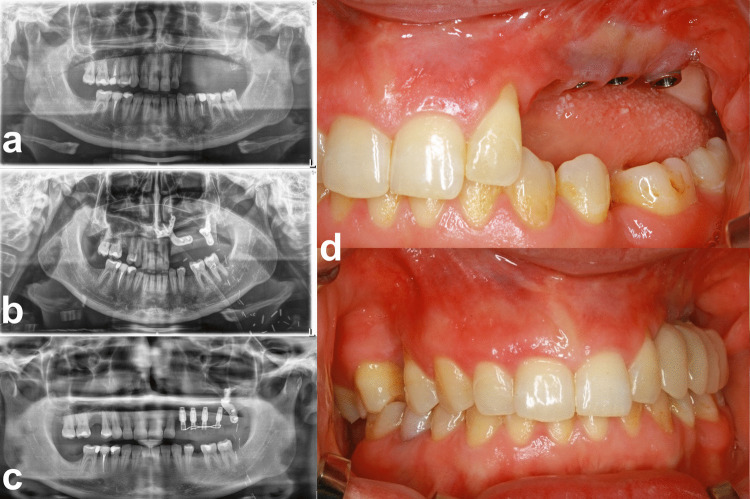
Patient with FFF flap reconstruction and secondary vestibuloplasty using split skin (**a** – preoperative panoramic X-ray; **b** – postoperative condition after three days; **c** – postoperative condition after six years; **d** – clinical condition after dental rehabilitation)

**Fig. 3:**
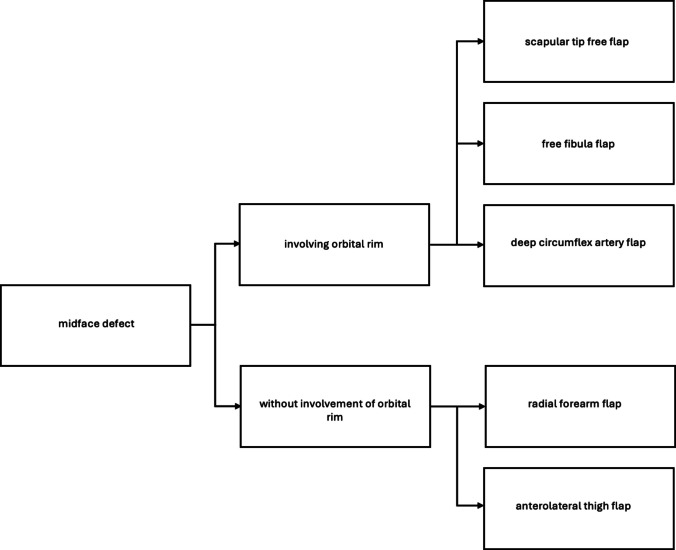
Midface reconstruction algorithm

Overall, this study represents the clinical practice of our clinic and substantiates it with a relevant number of cases. Our clinical experience indicates that the vertical dimension of midface defects is the most critical factor in determining the optimal reconstructive approach in cases of extensive defects, particularly where resection of the alveolar ridge or zygomatic bone is necessary, we continue to advocate for bony reconstructions in order to achieve an adequate aesthetic soft tissue profile. Depending on the extent of bony reconstruction required, free fibula flaps (FFF), deep circumflex iliac artery (DCIA) bone flaps (Fig. 2), or scapular tip flaps represent valuable options. Where bony reconstruction is not required, soft tissue flaps—particularly radial forearm flaps (RFF) or anterolateral thigh (ALT) flaps—are our preferred choice. Combinations of soft tissue free flaps with delayed preprosthetic implants may also prove beneficial. Taking these considerations into account, we recommend the following algorithm for midface reconstruction (Fig. 3):

This algorithm is explicitly grounded in our clinical experience and is intended as a pragmatic aid to decision-making rather than as a validated predictive model. It emphasises the vertical dimension of the defect as the most critical determinant, particularly in cases involving resection of the alveolar ridge or zygomatic bone. In addition, it incorporates the concept of delayed pre-prosthetic implant placement in selected cases reconstructed with soft tissue flaps, thereby offering an alternative to primary osseous reconstruction where appropriate.

However, formal validation of reconstructive algorithms in midfacial reconstruction remains challenging and is rarely undertaken in prospective comparative studies. Even in high-volume oncological maxillary reconstruction series, algorithmic frameworks are typically derived from retrospective cumulative experience across multiple surgeons and are often shaped by individual familiarity with specific flap techniques, thereby limiting their generalisability and reproducibility. The literature further highlights that variation in flap selection is frequently attributable to surgeon-specific preferences, reflecting the broader observation that such preferences may influence both indications and outcomes.

Chen et al. proposed a reconstruction algorithm based on four key anatomical structures of the midface: the anterior maxillary arch, the orbital floor, the globe, and the presence of oro-sinonasal communication. In their framework, loss of the anterior maxillary arch or the orbital floor with preservation of the globe constituted the principal indications for fibula free flap reconstruction. Where these structures could be preserved, they favoured the use of anterolateral thigh flaps [22].

Similarly, Hanasono et al. developed an algorithm focusing on palatoalveolar and orbital floor defects, with particular emphasis on the integrity of the orbital floor and orbital contents. Based on their experience with nearly 250 midfacial reconstructions using microvascular flaps, they advocated osseous reconstruction in cases involving the alveolar maxilla, while posterior defects could be managed with soft tissue flaps. Reconstruction of the orbital floor was considered essential when orbital contents were preserved, requiring rigid support with either bone or alloplastic materials. In cases of orbital exenteration, however, soft tissue reconstruction alone was deemed sufficient [23].

Both algorithms share a common principle: the decision to pursue bony reconstruction is primarily driven by the involvement of key osseous structures of the midface. This aligns with our clinical experience and is consistent with the rationale underlying our proposed approach. However, our algorithm extends this concept by incorporating the possibility of dental rehabilitation without primary osseous reconstruction. Specifically, it introduces the combined use of soft tissue flaps and delayed pre-prosthetic implant strategies as an alternative in anatomically suitable cases, with the aim of reducing the number of bony reconstructions that do not ultimately result in implant-based rehabilitation.

Overall, the study found a relatively high complication rate of 58.2%. Minor complications, including bleeding, infection, and wound healing disturbances, were common across all flap types and defect classes, whereas major complications were less frequent, with the need for flap revision in nine cases (9.2%) and flap loss in seven cases (7.2%). These results are comparable to the wide range of complication and flap loss rates reported in the literature. In agreement with our findings, major complications such as flap revision or loss occur infrequently, while minor complications are more common [5, 24–27]. Additionally, the malignant nature of midfacial defects may contribute to the relatively high complication rate observed.

Postoperative flap monitoring is crucial for identifying potential vascular complications and addressing them surgically if necessary. The first 48–72 h following surgery are particularly critical for short-term vascular complications [28]. At our centre, flap monitoring is conducted through clinical examination and circulation assessment, primarily using a pocket Doppler device. This monitoring is performed every two hours for the first three postoperative days and every four hours for the following four days. Additionally, anticoagulants are administered to reduce the risk of thrombotic events.

Diabetes mellitus is a well-documented risk factor for postoperative wound healing complications, increasing the likelihood of haemorrhage, infection, and necrosis [29–31]. However, our study did not find a significant association between diabetes and flap complications or failure. Similarly, alcohol consumption and smoking were evaluated for their impact on flap complications, but neither demonstrated a significant effect on flap outcomes. These findings are consistent with some reports in the literature [26]. It is important to acknowledge that our data on alcohol consumption and smoking rely on patient-reported information documented in digital records, which may underestimate actual consumption. This discrepancy could explain why other studies suggest an association between these factors and increased flap complications [32, 33].

Furthermore, body mass index (BMI), defect classification, and flap type did not significantly influence surgical outcomes in this study. Thus, appropriate flap selection appears to be more critical for the success of flap surgery than the patient’s general health condition. Moreover, rigorous flap monitoring plays a vital role in detecting complications at an early stage and preventing their progression into major complications.

Cordeiro et al. propose a flap selection algorithm based on their surgical experience, recommending the use of RFFs, either as skin or osteocutaneous flaps, for small defects, and rectus abdominis flaps for larger defects [6]. Brown and Shaw similarly advocate for RFFs in small defects but prefer deep circumflex iliac artery bone flaps for bony reconstructions [8]. Other authors favour the use of FFFs for maxillary reconstruction due to their long pedicle, ability to provide skin, bone, and muscle in a single flap, and their suitability for dental implant placement [5]. Nevertheless, the precise indications for soft tissue-only reconstruction versus additional bony reconstruction remain somewhat ambiguous. The choice must consider the surgeon’s expertise and preferences, as well as the patient’s overall condition and reconstructive goals. For instance, in cases where there is no strict medical indication or where the patient does not wish to undergo dental rehabilitation, bony reconstruction may not be necessary. Additionally, we observed higher rates of complications and flap failure when using bony flaps for the reconstruction of maxillary defects. Furthermore, in many patients who received bony flaps, dental rehabilitation was either not feasible or not undertaken.

The available literature highlights two principal observations regarding implant rehabilitation following oncological jaw surgery. First, the overall rate of implant-based dental rehabilitation appears to be relatively low, particularly in the maxilla, where data remain limited. Bousdras et al. reported an implant rehabilitation rate of 19.8% following maxillary or mandibular reconstruction using bony free flaps, a finding that closely mirrors the rates observed in our cohort. Notably, among those patients who were able to receive implants, outcomes were highly favourable, with 98.6% of implants achieving successful osseointegration [34]. Mücke et al. reported similar findings, with an implantation rate of 35.7% among patients undergoing bony microvascular reconstruction using fibula, scapula, or iliac crest free flaps. However, only a small proportion of these patients ultimately underwent complete dental rehabilitation, corresponding to an overall rehabilitation rate of 12.0% [35].

Dholam et al. investigated a cohort of 138 patients who underwent oncological jaw resection and bony reconstruction, of whom only 30% were considered suitable candidates for dental implants. The limiting factors included the morbidity associated with radiotherapy, interference from reconstruction plates and screws, unfavourable maxillomandibular relationships, and obliteration of the vestibule [36]. Similarly, Oldén et al. in a cohort of 41 patients undergoing mandibular reconstruction with fibula free flaps, reported that only 11 patients (27%) received dental implants, and just eight (20%) completed rehabilitation with an implant-supported prosthesis. Patient mortality and tumour recurrence were the primary reasons for failure to progress to the implant stage [37]. Comparable results were reported by Chiapasco et al., who found that only 27.1% of patients in a cohort of 59 undergoing maxillary or mandibular reconstruction with bony free flaps proceeded to implant placement [38].

Taken together, these findings support the conclusion that the overall rate of dental rehabilitation following bony maxillary reconstruction remains relatively low. This raises questions regarding the role of bony reconstruction as a foundation for implant-based rehabilitation. However, in those patients who do proceed to implant placement, outcomes appear to be consistently favourable.

In addition to implant rehabilitation, functional support of the midfacial soft tissues represents a critical consideration in reconstructive planning. Wu et al. therefore recommend bony reconstruction in cases involving the infraorbital rim or premaxilla, which is in line with the recommendations derived from our data [39].

**Fig. 4:**
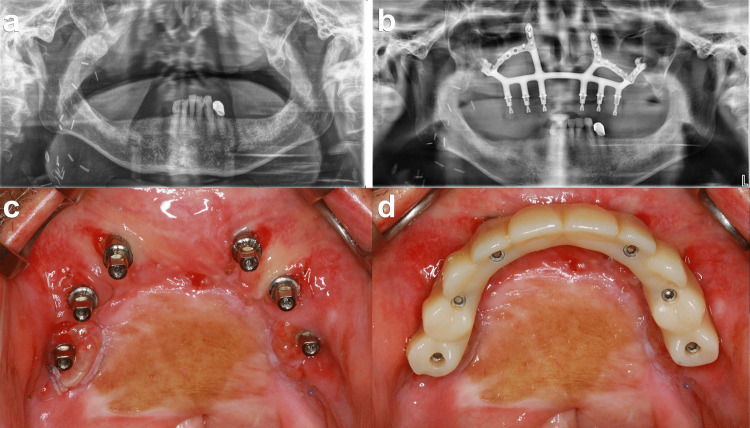
Patient with a preprosthetic maxillary implant after latissimus dorsi myocutaneous flap. (**a** – preoperative condition; **b**—**d** – postoperative condition)

Based on these findings, we recommend considering alternative approaches, such as combining preprosthetic implants with soft tissue flaps for reconstruction. This strategy leverages the advantages of CAD/CAM planning to create predictable clinical conditions for dental rehabilitation, while potentially reducing the higher complication rates associated with bony transplants. Furthermore, the insertion of preprosthetic implants can be performed on an outpatient basis and has the potential to reduce the effort required for dental rehabilitation with conventional implants in cases of large defects by eliminating the need for bone augmentation, the osseointegration period, and possible preprosthetic procedures such as vestibuloplasty. (Fig. 4).

One major limitation of this study is its retrospective design. As such, causal inferences cannot be reliably drawn. Furthermore, this study design is inherently vulnerable to incomplete capture of clinically relevant variables and outcomes, as no prospective clinical re-evaluation was performed. Subclinical or asymptomatic events may therefore not be consistently documented. Selection bias represents a further important limitation when interpreting differences between bony and soft tissue reconstructions in this cohort. Flap selection is often influenced by surgeon preference, particularly in the absence of standardised or protocol-driven allocation. In the present cohort, bony flaps constitute a significantly smaller subgroup than soft tissue flaps, increasing susceptibility to imbalance in baseline defect characteristics and other unmeasured confounders relevant to reconstructive strategy. Information bias is also a concern, as is typical in retrospective studies, particularly regarding patient-related factors such as alcohol consumption and smoking. These variables may be underreported, potentially leading to underestimation of their true extent. This is especially relevant given the heterogeneous comorbidity profiles observed in patients with head and neck malignancies, which may confound associations between exposure variables and postoperative outcomes.

Additional limitations relate to the rationale for selecting osseous flaps in the context of relatively low downstream rates of dental rehabilitation. In the present study, only 18.2% of patients undergoing bony reconstruction proceeded to dental rehabilitation. This finding challenges the assumption that osseous reconstruction will necessarily facilitate implant-based rehabilitation and underscores the need for a more nuanced interpretation of the functional benefits provided by bony flaps. The uptake of dental implants may also be influenced by patient preferences and competing treatment priorities. In this context, our proposed algorithm explicitly considers alternative strategies, such as soft tissue free flaps combined with delayed pre-prosthetic implant placement in cases where osseous reconstruction is not strictly indicated. However, the present data do not allow for robust identification of patient-, disease-, or pathway-level barriers underlying the low rate of implant-based rehabilitation observed.

## Conclusion

Complications in free flap surgery following the resection of malignant midface tumours are common, though the majority are minor. Major complications, such as flap failure, are comparatively rare. The tendency for complications and flap loss is higher with bone flaps compared to soft tissue flaps. Given the relatively low number of dental rehabilitations using implants in patients with bony midface reconstructions, the indication for their use must be critically evaluated on an individual basis for each patient. Patient-related factors, including diabetes mellitus, alcohol consumption, smoking, and BMI, did not significantly influence surgical outcomes in this study. Similarly, neither defect classification nor flap type appeared to impact surgical success.

In summary, free flap surgery remains a reliable approach for reconstructing midfacial defects following tumour resection. The key determinants of success appear to be appropriate flap selection and diligent postoperative monitoring, both of which are essential in achieving favourable outcomes.

## Data Availability

The data that support the findings of this study are available from the corresponding author upon reasonable request.
